# Variation in Bacterial Community Structures and Functions as Indicators of Response to the Restoration of *Suaeda salsa*: A Case Study of the Restoration in the Beidaihe Coastal Wetland

**DOI:** 10.3389/fmicb.2022.783155

**Published:** 2022-04-25

**Authors:** Changfei He, Li Zheng, Jinfeng Ding, Wei Gao, Qian Li, Bin Han, Jingxi Li

**Affiliations:** ^1^Key Laboratory of Marine Eco-Environmental Science and Technology, First Institute of Oceanography, Ministry of Natural Resources, Qingdao, China; ^2^Laboratory for Marine Ecology and Environmental Science, Pilot National Laboratory for Marine Science and Technology, Qingdao, China; ^3^Institute of Marine Science and Technology, Shandong University, Qingdao, China; ^4^Institute of Coastal Environmental Pollution Control, Ministry of Education Key Laboratory of Marine Environment and Ecology, College of Environmental Science and Engineering, Ocean University of China, Qingdao, China

**Keywords:** wetland restoration, bacterial community, biogeochemical functions, environmental factors, biological indicator

## Abstract

Microbes play an essential role in the restoration of degraded coastal wetlands. However, few studies have focused on the role of key bacteria in the restoration process. Here, *Suaeda salsa* was planted to recover the biodiversity in the degraded Beidaihe coastal wetland. We monitored omics and soil environmental factors to understand the complex relationship between the bacterial community and wetland health during the restoration period. After planting *S. salsa* in the degraded area, the soil quality was improved in the later stage of restoration (LPR). Bacterial α-diversity increased with restoration and was positively correlated with TOC. Proteobacteria is the dominant bacterial phylum during the restoration period, and Bacteroidetes, Planctomycetes, Gemmatimonadetes, and Acidobacteria were sensitive to the planting restoration. Random forest analysis picked 30 key OTUs, showing the key bacterial variation of successful restoration. The result indicated that the sum of the relative abundances of key bacterial OTUs was more than 2% in the health wetland. The β-diversity showed that the growth of *S. salsa* reshaped the soil bacterial community structure and function in the LPR, which recovered to the level in the control area. Putative biogeochemical functions showed that symbionts and aromatic compound degradation were dominant bacterial functions in the growth period of *S. salsa*. Our study proposed a new indicator to assess wetland health and the planting restoration of *S. salsa* increased bacteria groups with the ability of symbionts and aromatic compound degradation in the Beidaihe coastal wetland. This study expanded our knowledge of coastal wetland restoration and its ecological contributions.

## Introduction

Coastal wetlands are transitional gradients between terrestrial and oceanic ecosystems and play essential roles in biogeochemical cycles and biodiversity (Mcleod et al., 2011). Due to high primary production, coastal wetlands can produce 40% more plant biomass annually than the same area of forest (Bertness et al., 2008; Hopkinson et al., 2012). This means that coastal plants play an essential ecological role in response to carbon cycling in the world. Recently, an increasing number of coastal wetlands have undergone degradation and shrinkage due to marine pollution, such as oil spills and wastewater discharge. More than 50% of coastal wetlands are on the edge of degradation worldwide (Davidson, 2014). Wetland degradation causes the death of coastal vegetation (Dai et al., 2013), which dramatically decreases the net primary productivity and accelerates the salinization or barren of coastal soil. Replanting coastal vegetation is an efficient way to recover wetland health (Williams and Faber, 2001; Zou et al., 2014). For example, replanting mangroves in the degraded or damaged wetlands successfully recovered the ecological function of the coasts of southern Louisiana (Madison et al., 2013).

Due to a limited understanding of wetland restoration, it is still a challenge to restore degraded wetlands. Since the 1960’s, scientists have paid attention to coastal ecosystem degradation, and great efforts have been made to restore and recreate damaged ecosystems (Daily, 1995; Zhao et al., 2016). Although many coastal wetland restoration projects are conducted every year, wetland degradation has not been retarded worldwide (Kentula, 2000; Lv and Liu, 2008). Bacteria play a crucial role in the restoration process of degraded coastal wetlands. Understanding the bacterial roles in the restoration period enables us to understand further the degradation and restoration of coastal wetlands.

The bacterial community is sensitive in response to the variation of the habitat environment. In coastal wetland ecosystems, the soil is an important medium for interacting bacteria and various habitats (Mitsch et al., 2013). Bacteria grow in the soil, and any soil property will influence the community structure, such as salinity, total organic carbon (TOC), and heavy metals (Dupont et al., 2014; Beattie et al., 2018; Jílková et al., 2021). Heavy metals, usually toxic environmental pollutants, decrease biodiversity in a coastal wetland. In addition, coastal plants can absorb salts from the soil to decrease the salinization of coastal soil (Bernstein, 1975), or provide a carbon source for soil bacteria via phytodetritus and root exudates (Giere, 2009; Geisseler et al., 2011). Chaudhary et al. (2018) reported that the growth of halophytes increased the bacterial diversity and shaped the bacterial community structure in salt marshes.

The bacteria community is a potential biological indicator to assess the restoration effect and wetland health since they play mainly ecological roles in the material cycles, energy flow, and ecosystem stability during the restoration period (Yu et al., 2012; Lv et al., 2016; Ma et al., 2016). The abundance of *Desulfovibrio* decreased with the planting restoration in the Yellow River Delta, China (Ma et al., 2016). Biological indices, such as richness, minor populations of soil microbial communities, and abundance of microbes, have also been used to evaluate the restoration effects of wetlands in the coastal wetlands (Wortley et al., 2013; Zhang et al., 2015; Ma et al., 2017; Lee et al., 2020).

Thousands of different operational taxonomic units (OTUs) can be obtained, and the changes of the complex bacterial communities were illuminated by 16S rRNA sequencing analysis (Ma et al., 2016; Chaudhary et al., 2018; Jílková et al., 2021). Random forest (RF) models are an ensemble learning method for classification and regression that operates by constructing a multitude of decision trees at training time and outputting the class (Ließ et al., 2012). The key bacterial OTUs that responded to planting restoration might be picked using the machine learning method to assess the restoration effect. For example, the key bacterial OTUs were studied by RF modules to predict the plant ages during the rice life cycle (Edwards et al., 2018).

Since the Penglai 19–3 oil spill event occurred in 2011, half the *S. salsa* wetland had gradually degraded in the Beidaihe coastal wetland after a few years. In 2017, the planting restoration project was performed in the degraded area by the local government. We monitored the variation of soil environmental factors and bacterial community composition and functions at different time points during the restoration period. This study aims to: (i) understand the effects of planting restoration on soil environmental variables, bacterial diversity, composition, and function. (ii) establish new bioindicators via the soil bacterial community to assess or diagnose the health of coastal wetlands. (iii) explore the relationship between bacterial community composition and function and environmental factors during the restoration period.

## Materials and Methods

### Research Site

**FIGURE 1:**
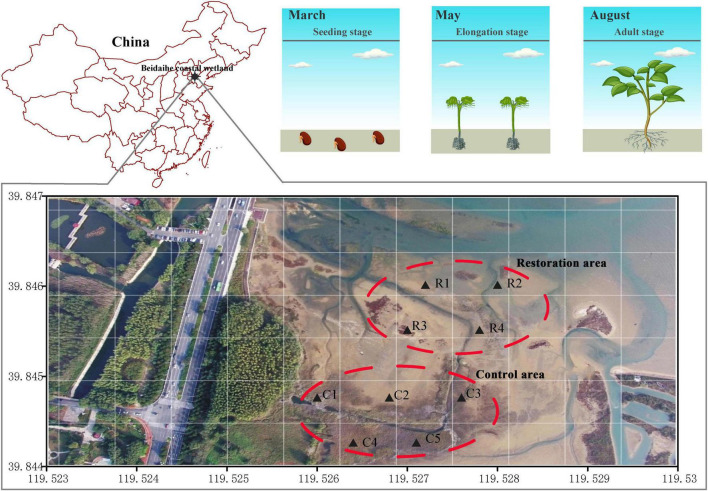
Geographic location of sampling area in the Beidaihe coastal wetland. The aerial picture was taken in August 2016. *Suaeda salsa* barely grew in the restoration area and was dense in the control area. We sowed *S. salsa* seeds in March 2017 in the restoration area. The control area did not suffer degradation.

The Beidaihe coastal wetland is located west of Bohai Bay, China, in a temperate monsoon climate zone, and the average annual precipitation is 530–630 mm, with 70% rainfall during the summer (May–July). *Suaeda salsa* is the dominant native vegetation, which begins to germinate in March, elongates in May, and flourishes in August. Owing to the oil spill contamination in 2011, half of the coastal wetland had degraded in our restoration area (approximately 7.3 hectares). *Suaeda salsa* was planted to restore the vegetation in the degradation area. Four sites (R1–R4) were established to research the soil bacterial community during the restoration period. The other half of the coastal wetland was not degraded, in which the *S. salsa* has been growing well, as the control area. Five sites (C1–C5) were set up to study the soil bacterial diversity (Figure 1).

In the restoration area (R1–R4), *S. salsa* was planted seeds in March. To improve the density of *S. salsa* in the restoration area, *S. salsa* seedlings was replanted in May again. In August, *S. salsa* grew exuberantly in the restoration area and the *S. salsa* coverage reached approximately 80% compared to the control area.

### Sample Collection

There are nine trial sites to collect the samples. Three replicates of surface soil (0–5 cm) were randomly collected at each site to be assemble as a mixed sample, which was used to extract genomic DNA and detect environmental parameters. A total of 27 soil samples were collected in March, May, and August, respectively (Supplementary Table 1). Based on sampling sites and collection time, 27 samples were divided into six subsamples, including EPR (Early Period of Restoration, the soil samples in the restoration area in March before restoration started), EPC (Early Period of Control, the soil samples in the control area in March), PR (Period of Restoration, the soil samples in the restoration area in May), PC (Period of Control, the soil samples in the control area in May), LPR (Later Period of Restoration, the soil samples in the restoration area in August), and LPC (Later Period of Control, the soil samples in the control area in August).

### DNA Extraction, PCR Amplification, and Sequencing

According to the manufacturer’s instructions, DNA was extracted from 0.5 g of soil sample using a PowerSoil DNA isolation kit (MoBio, Carlsbad, CA, United States). Extracted DNA was quantified using a spectrophotometer (Nanodrop, PeqLab, Germany). The V3-V4 region of bacterial 16S rRNA genes was amplified from the total DNA by PCR, using the specific primers 341F: CCTACGGGNGGCWGCAG and 806R:GGACTACHVGGGTATCTAAT (Guo et al., 2017). with sample-specific barcodes. The Illumina PE250 library was constructed and sequenced at Novogene Bio-Technologies Co., Ltd., Tianjin, China.

The 16S rRNA gene sequences were processed using QIIME 1.9.1, USEARCH (Caporaso et al., 2010; Bokulich et al., 2013). Raw reads with an average phred score of < 20 were discarded, and a 10-bp window from the first base with a 1-bp step length was used to filter. The detailed results showed in Supplementary Table 2. The rarefaction plots were presented in Supplementary Figure 1. The clean paired-end Illumina reads were joined, extracted by the join_paired_ends.py and extract_barcodes.py scripts. Based on the high-quality 16S representative sequences at 97% identify level, an OTU table was generated by USEARCH (Edgar, 2010). The representative sequences were conducted using the RDP classifier (Version 2.2) and annotated with the SILVA 138.1 (Pruesse et al., 2007; Wang Q. et al., 2007; Quast et al., 2013). Functional annotation of prokaryotic taxa (FAPROTAX) is a manually constructed database that maps prokaryotic taxa to putative functions based on the literature on cultured representatives (Louca et al., 2016). A Python script, collapse_table.py,^1^ can convert the OTU tables into putative functional tables based on the taxa identified in a sample and their functional annotations in the FAPROTAX database. The bacterial taxon of each function was obtained from a report file.

### Statistical Analysis

All statistical analyses and plots were performed using R software (version 3.6^2^) (Ginestet, 2011). The Shannon and Simpson diversity was calculated using the “diversity” function, and the “rda” function conducted redundancy analysis (RDA) for linking bacterial communities to environmental variables using the Vegan package (Oksanen et al., 2013). The Monte Carlo permutation test (permu = 999) was performed to detect the significance of the environmental variables. Analysis of differential phylum abundance was performed using a linear model in the Performance Analytics package (Peterson et al., 2014). Analysis of variance (ANOVA) was performed using the “aov” function from the Stats package (R Core Team, 2018). A “cor” function calculated the Spearman correlation coefficient, and the p-value was adjusted with “p.adjust” (method = “bonferoni”) in the R.

### Generation of Sparse Random Forest Models

To model the health of wetland soil as a function of the bacterial community, we developed full RF models for soil samples by regressing the relative abundance of all OTUs against the healthy state of coastal wetland soil from which the samples were collected. For the training data, we selected eight samples from March and August in the degradation region. From the model, we ranked individual OTUs by their importance in contributing to the accuracy of wetland health prediction by the model. This process was performed by permuting the relative abundance levels for an OTU and calculating the increase in the mean squared error of the model. When permuted yield increased errors in the model, the OTU abundance was essential to the model’s accuracy. The step was performed using the “importance” command from the random Forest R package (Liaw and Wiener, 2002). Because not all OTUs in the RF model contributed to the accuracy of the model, we next performed 10-fold cross validation to evaluate model performance using the “replicate” function in the randomForest R package. We found a minimal increase in accuracy when including more than 30 of the most important OTUs (Supplementary Figure 2). The top 30 important OTUs from the full RF model were used as input for sparse RF models for each phase.

### Measurements of Environmental Parameters

Soil salinity was measured according to the method of Gartley (2011). Briefly, aqueous extract (mix 50 mL water with 10 g air-dried soil) of soil sample was prepared by shaking the mixture for 5 min and then allowed to settle for 4 h. The extraction was used to detect soil salinity with a conductivity meter (YSI Incorporated Ohio). 15 g of soil was weighed and dried to constant weight using a vacuum freeze drier (24 h) to detect the soil water content. A gas chromatography-mass spectrometer (GC-MS) was utilized to detect the polycyclic aromatic hydrocarbon (PAHs) concentration, according to Lin et al. (2018). Briefly, a total of 5 g air-dried soil (filtered through 100 mesh screen) was extracted twice with N-hexane/dichloromethane solution (1:1, v/v). The extract solution was dehydrated by anhydrous sodium carbonate and filtered through a cellulose acetate membrane (0.2 μm). The solution was used to detect the PAH concentration with 5973N GC-MS (Agilent, United States). Total organic carbon (TOC) in the soil samples was detected with a Vario Micro Cube Elemental Analyzer (Elementar, Germany) (Sun et al., 2014). The heavy metal contents were determined using ICP-MS (Agilent ICP-MS 7500a) (Li et al., 2017). The steps were as follows: the soil samples were ground to a fine powder using a pestle in an agate mortar after drying. Powdered samples weighing 0.1 g were digested by a microwave system. The digestion solution was fixed to 25 g with ultrapure water to detect the concentration of heavy metals. Meanwhile, 5 μg/L Re element was considered as the internal standard element. The heavy metal contents in certified reference materials (Yellow Sea marine sediments, GBW07333) were measured to verify the accuracy and precision of the analytical method.

## Results

### The Alpha Diversity of Bacteria and Soil Physicochemical Properties

During the restoration time in the Beidaihe coastal wetland, a total of 10386 different OTUs were identified. In the restoration area, bacterial richness gradually increased with restoration (EPR: 0.17, PR: 0.21, LPR: 0.41). The richness slightly increased with time in the control area (EPC: 0.23, PC: 0.23, LPC: 0.33). In the later period of restoration (LPR and LPC), the bacterial diversity was significantly high compared to other periods (Supplementary Figure 3). A similar trend was shown in OTU numbers, shannon index and simpson in the Beidaihe coastal wetland (Table 1 Part A).

**TABLE 1:** Alpha-diversity and environmental factors in different restoration periods between degradation and non-degradation regions.

Site	OTUs	Shannon	Simpson	Water Content (%)	Salinity (g/kg)	PAHs (ng/g)	TOC (%)	Cr (mg/Kg)	Cu (mg/Kg)	Zn (mg/Kg)	As (mg/Kg)	Cd (mg/Kg)	Pb (mg/Kg)
		
Part A				Part B				Part C					
EPR	^b^ 3614	^b^5.72	0.989	0.18 ± 0.01	2.45 ± 0.67	^b^1875.29 ± 310.24	^b^0.17 ± 0.01	10.4 ± 1.09	4.51 ± 0.81	13.20 ± 2.46	^a^1.96 ± 0.3	0.06 ± 0.01	10.13 ± 0.71
EPC	^b^ 5318	^b^6.00	0.992	0.18 ± 0.01	2.4 ± 0.45	^b^1606.3 ± 130.31	^b^0.23 ± 0.04	11.6 ± 1.77	4.28 ± 0.85	12.04 ± 1.53	^a^2.01 ± 0.28	0.03 ± 0.01	11.45 ± 2.34
PR	^b^ 4528	^b^6.00	0.993	0.21 ± 0.02	1.63 ± 1.09	^b^1490.61 ± 165.3	^b^0.26 ± 0.11	11 ± 2.08	5.51 ± 0.94	14.23 ± 2.67	^b^1.67 ± 0.14	0.06 ± 0.01	9.54 ± 0.92
PC	^b^ 5648	^b^6.04	0.987	0.22 ± 0.01	2.16 ± 0.54	^b^1435.63 ± 382.15	^a^0.48 ± 0.11	10.72 ± 0.6	5.08 ± 0.77	12.75 ± 1.82	^b^1.82 ± 0.1	0.03 ± 0.01	10.14 ± 2.32
LPR	^a^ 6935	^a^ 6.69	0.994	0.19 ± 0.04	1.23 ± 0.39	^a^694.95 ± 8.83	^a^0.52 ± 0.11	9.20 ± 2.4	4.44 ± 0.76	12.71 ± 1.24	^b^1.44 ± 0.09	0.03 ± 0.01	9.56 ± 2.31
LPC	^a^ 6608	^a^6.46	0.993	0.18 ± 0.03	1.79 ± 0.25	^a^1016.62 ± 169.10	^a^0.55 ± 0.11	9.82 ± 1.9	3.81 ± 0.86	10.80 ± 1.92	^b^1.80 ± 0.13	0.02 ± 0.01	7.63 ± 0.47

*Part A, Alpha-diversity of bacteria in different groups. Part B, the environmental parameters in different groups. Part C, the heavy metal concentration in different groups. The Kruskal–Wallis test was used to determine the significant difference:*

*^a,b^ the different letters represent the significant difference (p < 0.05) among all soils.*

The soil environmental factors and heavy metal concentrations are shown in Table 1 Part B and Part C in the restoration period of Beidaihe coastal wetland. The soil water content ranged from 18 to 22%, slightly high in PC and PR (May). The soil salinity decreased slightly during the growth of *S. salsa*. The highest soil salinity (2.45 ± 0.67 g/kg) was measured in the EPR, and the lowest (1.23 ± 0.39 g/kg) was measured in the LPR. The PAH concentration in LPR (694.95 ± 8.83 ng/g) and LPC (1016.62 ± 169.10 ng/g) was significantly lower than that in EPR (1875.29 ± 310.24 ng/g), EPC (1606.3 ± 130.31 ng/g), PR (1490.61 ± 165.3 ng/g), and PC (1435.63 ± 382.15 ng/g). The TOC concentration was significantly higher in PC, LPR, and LPC than in EPC, EPR, and PR, showing that the growth of *S. salsa* introduced the organic carbon to coastal soil. Among the heavy metals, the order of concentration was Zn (10.8 – 14.23 mg/kg) > Cr (9.2 – 11.6 mg/kg) > Pb (7.63 – 11.45 mg/kg) > Cu (3.81 – 5.51 mg/kg) > As (1.44 – 2.01 mg/kg) > Cd (0.02 – 0.06 mg/kg). In general, the concentration of all heavy metals showed a slight decrease during the wetland restoration process. The heavy metal, As decreased significantly in PR, PC, LPR, and LPC.

**FIGURE 2:**
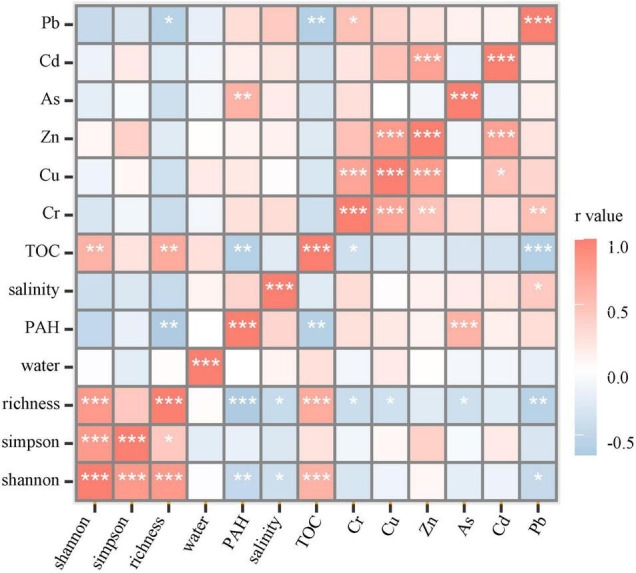
The spearman correlation in bacterial diversity and soil environmental factors. The * stands for the *p* value. *, *p* < 0.05; **, *p* < 0.01; ***, *p* < 0.001.

The spearman correlation showed that bacterial richness and the shannon index significantly positive correlated with the TOC concentration and negatively correlated with heavy metals, PAHs, and salinity. A significantly negative correlation was shown between TOC concentration and some contaminants, such as PAHs and heavy metals, Cr and Pb (Figure 2). The relationship illustrated that bacterial diversity increased in response to the high TOC concentration but decreased due to the high PAH, heavy metal concentration, and salinity in the soil.

### Bacterial Community Composition During the Restoration Time

**FIGURE 3:**
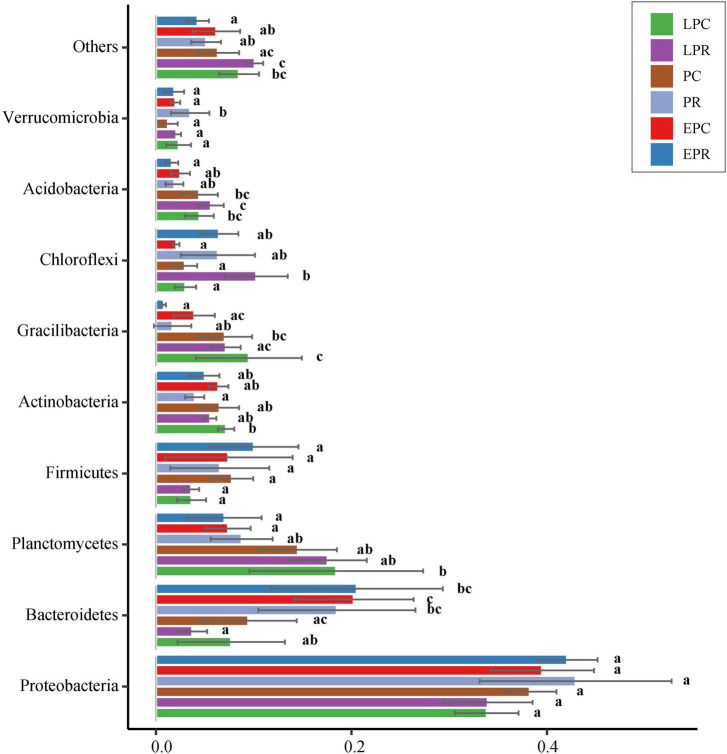
Bar plots of the top 9 phyla abundances during different restoration periods in the degraded and non-degraded wetland. ^a, b, c^The different letters represent the significant difference (*p* < 0.05) between different groups.

A total of sixty-nine different phyla were discovered during the restoration time. The bacterial communities were dominated by the phylum Proteobacteria, followed by Bacteroidetes, Planctomycetes, Firmicutes, Actinobacteria, Gemmatimonadetes, Chloroflexi, Acidobacteria, and Verrucomicrobia, which accounted for 94% of all bacterial reads (Figure 3). Correlation analysis showed that the relative abundance of Bacteroides presented a significantly negative correlation with that of Planctomycetes, Gemmatimonadetes, and Acidobacteria (Supplementary Figure 4).

Proteobacteria were dominant in restoration period, and the relative abundance decreased slightly with the growth of *S. salsa* (Figure 3). Gammaproteobacteria, Alphaproteobacteria, and Deltaproteobacteria were the abundant classes of Proteobacteria. Deltaproteobacteria were more abundant in the restoration area than that in the control area. Epsilonproteobacteria were the most abundant in EPR, and the relative abundance decreased with the growth of *S. salsa* in the restoration area (Supplementary Figure 5). In Bacteroidetes, its relative abundance was lower in the PC, LPC, and LPR compared to that in EPC, EPR, and PR, showing that the growth of *S. salsa* likely decreased the relative abundance of Bacteroidetes. The dominant classes were Flavobacteriia, Bacteroidia, and Sphingobacteriia in the Bacteroidetes. The relative abundance of Flavobacteriia decreased gradually with restoration time in the degradation area and had highest abundance in the EPR. Bacteroidia and Sphingobacteriia were dominant in the control areas (Supplementary Figure 6). Relative abundance of Planctomycetes in LPC, PC, and LPR were higher than in EPC, EPR, and PR. Dominated classes, Planctomycetaceae and Phycisphaeraceae, accounted for more than 72% of the total reads in Planctomycetes (Supplementary Figure 7). For the Acidobacteria, its relative abundance was high in LPC, PC, and LPR. Holophagae was most abundant in Acidobacteria and mostly distributed in EPR and PR (Supplementary Figure 8).

The bacteria community varied at the genus level during the restoration time. The top ten genera in each sample were chosen to analyze the bacterial community (Supplementary Figure 9). These genera account for approximately 45% to 67% of all bacterial OTUs. In the restoration area, *Desulfosarcina*, *Ilumatobacter*, *Loktanella*, and *Actibacter* were dominant genera in EPR and PR and the abundances of these genera were significantly higher than those in other periods. The growth of *S. salsa* shaped the distribution of dominant genera at the restoration area. These genera include *Planctomyces*, *Exiguobacterium*, *Citrobacter*, *Rhodopirellula*, *Pir4_lineage*, *Urania-1B-19_marine_sediment_group*, *Blastopirellula*, *Acinetobacter*, *unidentified bacterium wb1_A18*, and *Pseudomonas*, were dominant bacteria in LPR and were consistent with the predominant genera in PC and LPC. It is worth noting that the bacterial community in EPC had uniquely dominant genera, *Gramella*, *Roseovarius*, and *Marinobacter*, the relative abundances of which were significantly higher than those in the other groups.

### The Key Bacterial Operational Taxonomic Units During the Restoration Time

Operational taxonomic units (OTUs) are the basic taxonomic units for bacterial community structure and are sensitive to the variation of *S. salsa* growth and environmental factors (Edwards et al., 2018; Liu et al., 2019). Based on the random forest (RT) model, a total of 30 different key bacterial OTUs were identified during the restoration time, which could successfully predict 73.7% of the test samples (Supplementary Table 3), showing that these key OTUs can be used to represent the variation of the bacterial community during the restoration process.

**FIGURE 4:**
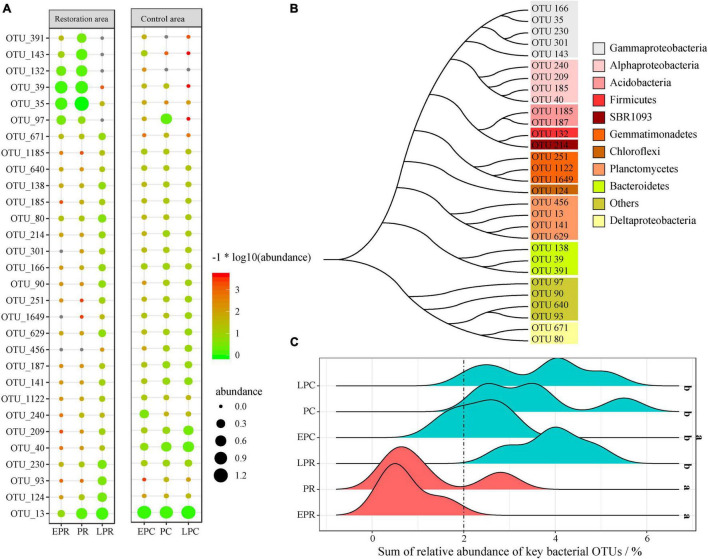
The random forest (RF) model detects the key operational taxonomic units (OTUs) during the restoration time. **(A)** The fingerprint spectrum of key OTUs in different groups; **(B)** phylogenetic analysis of key OTUs; **(C)** the sum of relative abundance of key OTUs in different groups. ^a, b^The different letters represent the significant difference (*p* < 0.05). OrangeRed represents the samples during the degradation period. Cyan stands for samples of restoration success and control area.

The relative abundance of the key OTUs in different groups is shown in Figure 4A. Many key OTUs were abundant in LPR, ECP, PC, and LPC. Based on V3-V4 sequences, the phylogenetic analysis found that these key OTUs were divided into 11 different clusters, including Gammaproteobacteria, Alphaproteobacteria, Acidobacteria, Firmicutes, SBR1093, Gemmatimonadetes, Chloroflexi, Planctomycetes, Bacteroidetes and Deltaproteobacteria (Figure 4B). Most key OTUs belonged to Proteobacteria, Planctomycetes, and Bacteroidetes. The detailed annotation results of each key OTU are shown in Supplementary Table 4. For example, OTU_391, OTU_143, OTU_132, OTU_39, and OTU_35 were annotated into *Winogradskyella*, *Marinobacter*, *Fusibacter*, *Sediminicola*, and *Halioglobus*, respectively, and these genera were most abundant in EPR and PR. OTU 13 belonged to Planctomyces and was the dominant OTU in LPR, EPC, PC, and LPC.

Among the relative abundance of key bacterial OTUs in the whole bacterial community, the sum of the relative abundances of all key bacterial OTUs was a potential biological indicator to evaluate the restoration effect and health (Figure 4C). The sum of the relative abundance of all key bacterial OTUs ranged from 0.8 to 5.7% during restoration time. However, it ranged from 2 to 5.7% in LPR, PC, and LPC, significantly higher than in EPR and PR (< 2%). It was noteworthy that the sum of the relative abundance of all key bacterial OTUs in EPC was not significantly different from that in the other groups.

### The Distribution of the Bacterial Community and Function During Restoration Time

**FIGURE 5:**
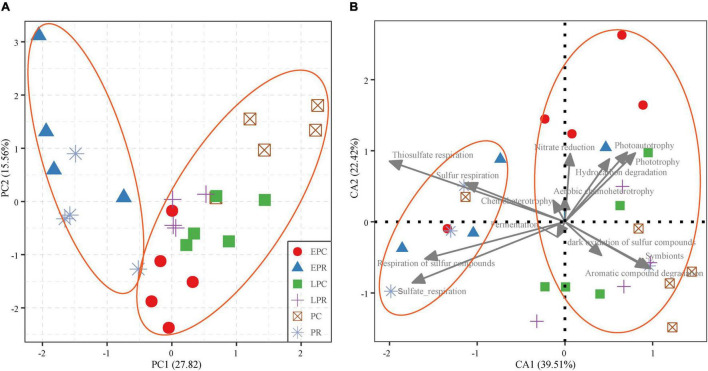
The β-diversity of the bacterial community **(A)** and bacterial community function **(B)**. The shape and color of the point stand the different groups. The gray line is the dominant bacterial function (the relative abundance > 1%).

The β-diversity of the bacterial community and function are shown in Figure 5. PCA results showed that the bacterial community in LPR was most similar to that in the control area, indicating that the growth of *S. salsa* shaped the bacterial community in the restoration area (Figure 5A). And the bacterial community function represented a similar variation with the community (Figure 5B). In the degraded periods (EPR and PR), bacterial community function was most related to the respiration of inorganic sulfur compounds, such as sulfur respiration, sulfate respiration, respiration of sulfur compounds, and thiosulfate respiration. However, the dominant bacterial functions included aromatic compound degradation, symbionts, hydrocarbon degradation, nitrate reduction, phototrophy, and photoautotrophy in the health periods (control and LPR) (Figure 5B). The β-Diversity analysis showed that the growth of *S. salsa* shaped the bacterial community in the soil and affected the bacterial community’s function.

### Bacterial Community Connects With Soil Environmental Factors During the Restoration Time

**FIGURE 6:**
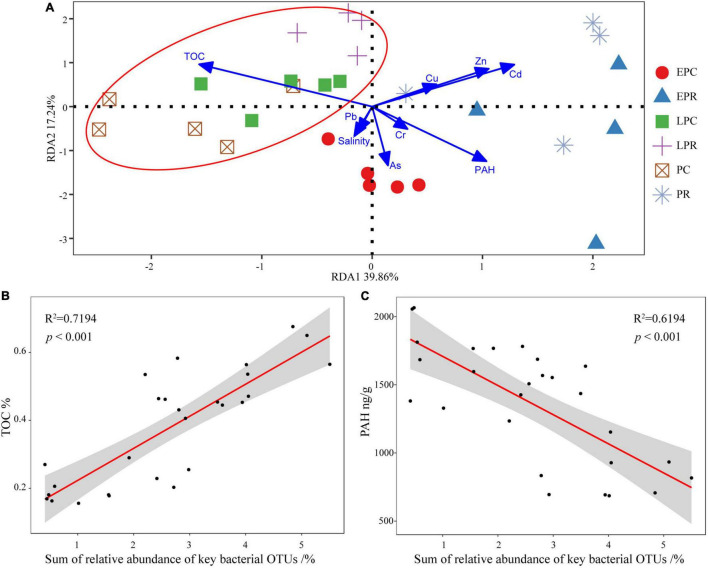
The relationship between bacterial community structure and environmental factors. **(A)** Redundancy analysis (RDA) analyze the relationship between bacterial community and environmental factors in different groups. The shape and color of the point stands for different groups. Linear-regression analysis between the sum of relative abundance of key operational taxonomic units (OTUs) and total organic carbon (TOC) **(B)**, and polycyclic aromatic hydrocarbon (PAH) **(C)** concentration.

To study the relationship between the bacterial community and soil environmental factors, redundancy analysis (RDA) was performed, and the first two axes explained 39.86 and 17.24% of the total variance, respectively (Figure 6A). The first axis was driven by soil properties (TOC, PAHs) and some heavy metals Cu/Zn/Cd, while the second axis was driven by salinity and As. The TOC concentration correlated significantly with the bacterial community structure in LPR, PC, and LPC. The concentrations of heavy metals and PAH significantly affected the community structure in EPR and PR. The bacterial community in EPCs could be affected by salinity and heavy metals (As).

As a biological indicator, the sum of the relative abundance of 30 key bacterial OTUs correlated closely to the variation in soil environmental factors. We found that the sum of the relative abundance of all key bacterial OTUs was significantly positively correlated with the TOC concentration (Figure 6B) and negatively correlated with the PAH concentration in the soil (Figure 6C). This showed that the sum of the relative abundance of all key bacterial OTUs was most sensitive in response to the various environmental factors.

### Bacterial Community Function and Association With Soil Environmental Factors During the Restoration Process

**FIGURE 7:**
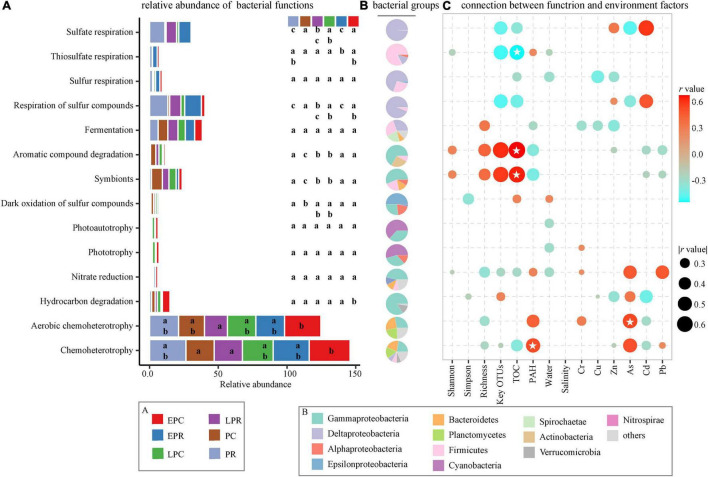
Bacterial ecological roles during the restoration time. **(A)** The relative abundance of different bacterial functions. The color presents different groups, consistent with the color in Figures 3, 5, 6. **(B)** The main bacterial phylum contributed to the ecological functions. The different colors stand for the bacterial taxon. **(C)** The connection between bacterial function and soil environmental factors. | r | > 0.3, * stands for *p* < 0.05.

Bacteria are involved in diverse ecological roles, including chemoheterotrophy, aerobic chemoheterotrophy, respiration of different S-containing substances, symbionts, hydrocarbon degradation, fermentation, aromatic compound degradation, and phototrophy (Figure 7). Among them, the respiration of inorganic sulfur compounds, such as sulfate respiration, sulfur respiration, respiration of sulfur compounds, and thiosulfate respiration, was significantly dominant in the restoration area compared to that in the control area, and the relative abundance decreased slightly in the LPR of the restoration area. Deltaproteobacteria and Firmicutes were involved in the respiration of inorganic sulfur compounds during the restoration time (Figure 7B). The ecological function of symbionts and aromatic compound degradation was determined via Gammaproteobacteria, Bacteroidetes, and Firmicutes. Theirs relative abundances were significantly higher in LPR, PC, and LPC than in EPR, PR, and EPC. Chemoheterotrophy and aerobic chemoheterotrophy were the most abundant bacterial community roles during the restoration time, showing that heterotrophic bacteria played an essential role in the material cycle of coastal wetlands.

Spearman correlation was applied to study the relationship between bacterial community function and soil environmental factors (Figure 7C). Bacterial diversity (richness), the sum of key OTUs, and TOC were positively correlated with symbionts and aromatic compound degradation functions and negatively related to the respiration of inorganic sulfur compounds during the restoration time. The PAH concentration and some pollutants were significantly positively correlated with the bacterial chemoheterotrophy and negatively associated with the symbionts and aromatic compound degradation. The relationship showed that the restoration of *S. salsa* promoted the growth of bacteria with symbionts and aromatic compound degradation functions to improve the soil TOC. Among the degraded area, bacteria with the respiration of inorganic sulfur compound function were abundant, negatively correlated to soil TOC to hold back organic carbon storage.

## Discussion

The degradation of coastal wetlands is an environmental problem worldwide. An increasing number of scientists are trying their best to treat this “environmental disease” (Williams and Faber, 2001; Zou et al., 2014; Sapkota and White, 2020). *Suaeda salsa* is a salt-tolerant plant (Lynum et al., 2020) and serves as the dominant native plant in Beidaihe coastal wetland, which is a suitable species to restore the degraded wetland in our restoration project. Previous restoration projects have successfully recovered the ecological function by planting *S. salsa* in China’s Yellow River Delta (YRD) (Ma et al., 2017). In our study, we traced the process of planting restoration and explored the soil environmental factors and bacterial community and function to understand the relationship between soil bacteria and *S. salsa* restoration.

The restoration of planting *S. salsa* improved the soil organic carbon concentration and changed the bacterial community in coastal wetlands. Bacteria can decompose the particulate matter to produce organic carbon in the soil, such as biological carbon pump theory in the ocean (Coleman, 1994; Jiao et al., 2018; Lian et al., 2021). In our study, soil TOC concentration was significantly higher in the LPR than in the EPR and PR (Table 1), indicating that the growth of *S. salsa* potentially provided the organic matter for soil microbial activities and shaped community structure and function. PCA analysis found that the bacterial community in LPR was more similar to that in the control area (Figure 5). In addition, *S. salsa* was a primary net primary productivity in Beidaihe coastal wetland, which can fix the carbon dioxide into organic carbon to be delivered into the soil by root exudates (Giere, 2009; Geisseler et al., 2011; Chaudhary et al., 2018; Ward, 2020). Our study showed that the growth of *S. salsa* increased the symbiotic function of the soil bacterial community. Symbiosis was any close and long-term biological interaction between two different biological organisms (Brinkman et al., 2002), suggesting a close exchange between bacteria and *S. salsa*.

Due to its biotoxicity, PAHs are an important and typical pollutant of oil contaminants (Vane et al., 2014). The Beidaihe wetland has been damaged by oil pollution since the Bohai 19–3 oil spill accident occurred in 2011. Lin et al. (2018) conducted a survey and ecological risk assessment of PAHs in this wetland in 2016. It was found that the ecological risk of PAHs in degraded regions reached a medium level. In our study, the PAH concentrations decreased gradually with restoration. Biological degradation was the main process of PAH transformation in the coastal wetlands (Bourceret et al., 2018). The aromatic compound degradation functions were abundant in LPR, PC, and LPC, suggesting that the growth of *S. salsa* promotes the microbial degradation of PAH, which can be explained by the soil priming effect (Bastida et al., 2019).

In addition, heavy metal elements (As, Cd, Cr, Zn, and Cu) affected the bacterial community structure due to a toxic effect, particularly at high concentrations (Wang Y. et al., 2007; Li et al., 2020). In our study, heavy metals decreased slightly with restoration, affected the bacterial community in EPR and PR, and were negatively related to bacterial richness and the Shannon index. Some studies have proven that *S. salsa* can absorb heavy metals from soil (Lutts and Lefèvre, 2015; Zhang et al., 2018). A previous study showed that some heavy metals negatively affected microbial biomass accumulation and productivity even at low concentrations, such as 1 ppm for Pb, 2 ppm for Cd and 5 ppm for Zn (Beattie et al., 2018).

The biological and chemical parameters had profitably changed in LPR, suggesting a successful restoration in the Beidaihe coastal. For example, the respiration of inorganic sulfur compounds was more abundant in the restoration area, and their relative abundance decreased slightly in LPR (Figure 7). Bacteria utilize the inorganic sulfur compounds as electron acceptors to produce reduced sulfur, such as hydrogen sulfide, via respiration (Hedderich et al., 1998; Florentino et al., 2016). Hydrogen sulfide is a broad-spectrum poison (Lindenmann et al., 2010), suggesting a wetland health threat of respiration of inorganic sulfur compounds in the restoration area.

The assessment and diagnosis of coastal wetland health are the most important for efficient management. In our study, the soil environmental parameters, bacterial community structure, diversity, and function shifted after the restoration of *S. salsa*, which were good indicators to evaluate the restoration effect. High TOC (Ruiz-Jaen and Aide, 2005; Milton and Finlayson, 2018) and low pollutant concentrations (Chen et al., 2019) indicated healthy soil quality, similar to the soil parameters in LPR (Table 1). Due to the limited information on monitoring data, these parameters lack efficiency and accuracy to define the restoration effect in coastal wetlands (Suding, 2011). Urakawa and Bernhard (2017) reported that the evaluation of restoration could not be met effectively by a single physical and chemical parameter, but a combination of multiple attributes is effective for robust wetland assessment and management. Therefore, bacterial populations serve as the most sensitive and rapid bioindicator in response to various environmental changes, which is suitable to evaluate wetland health. A study showed that bacterial richness could be applied to assess coastal wetland health (high richness was considered a healthy wetland) (Delgado-Baquerizo et al., 2016; Urakawa and Bernhard, 2017). In our study, the bacterial richness in LPR and LPC was significantly higher than that in EPR and PR but was not significantly different from that in PC and EPC, suggesting a low efficiency and accuracy to assess wetland health. Notably, the sum of key OTUs showed a significantly different in PC period, suggesting a higher accuracy compared to richness (Figure 4C). The sum of key OTUs closely related to the soil parameters (TOC and PAH concentration) and bacterial function (decreasing respiration of inorganic sulfur compounds and increasing symbionts and aromatic compound degradation during the restoration time), indicating that the sum of key OTUs was a synthetic attribute of multiple attributes in chemistry and biology. This presented a new bioindicator to assess or diagnose coastal wetland health, which was more efficient and accurate than other parameters.

In this study, our results provided a good reference for the health assessment of wetlands by key bacterial OTUs. Among the RF modules, it is difficult to control the inner workings of the model, like a “black box.” It is more helpful to improve the RF module by the large-scale samples and more attempts between different parameters and random seeds. In the future, Large-scale studies in the coastal wetland degradation will provide sufficient evidence for the key bacterial OTUs by RF modules as bioindicator to assess the restoration effect and wetland health. Meanwhile, the absolute abundance of key OTUs is necessary to understand the variation of key OTUs in the quantity level.

## Conclusion

The degraded Beidaihe coastal wetland was restored by planting native vegetation *S. salsa*. Based on the restoration processes, we found that the soil TOC and bacterial diversity increased, and pollutant concentrations, such as PAHs and heavy metals, decreased after the restoration of *S. salsa*. This indicated that restoration could potentially improve the carbon storage ability in the soil. In addition, variations in biological and chemical parameters can be applied to predict soil health, such as high soil TOC and bacterial diversity and low pollutant concentrations. Interestingly, we found that the sum of key OTUs is closely related to the soil TOC, PAH, and bacterial functions, and it can accurately evaluate the effect of the restoration. This case study is the first time to propose an accurate biological indicator to assess or diagnose coastal wetland health. Although our research was focused on northern China’s coastal wetlands, it provides a good reference for the health assessment of other wetlands.

## Data Availability Statement

The datasets presented in this study can be found in online repositories. The names of the repository/repositories and accession number(s) can be found below: https://www.ncbi.nlm.nih.gov/bioproject/PRJNA767015.

## Author Contributions

LZ and CH conceived the study. CH wrote the manuscript and performed the statistical analyses. LZ revised the manuscript. JD, WG, and QL involved in the field investigation and soil sampling. BH and JL performed PAHs and heavy mental analysis. All authors discussed the results and commented in the manuscript.

## Conflict of Interest

The authors declare that the research was conducted in the absence of any commercial or financial relationships that could be construed as a potential conflict of interest.

## Publisher’s Note

All claims expressed in this article are solely those of the authors and do not necessarily represent those of their affiliated organizations, or those of the publisher, the editors and the reviewers. Any product that may be evaluated in this article, or claim that may be made by its manufacturer, is not guaranteed or endorsed by the publisher.

## Footnotes

1
http://www.loucalab.com/archive/FAPROTAX/lib/php/index.php?section=Download

2
https://cran.r-project.org/

## Abbreviations

EPC: the early period in the control area. Samples include EPC1 to EPC5 in March
EPR: the early period of planting restoration in the degraded area. Samples includes EPR1 to EPR4 in March
PC: the planting period in the control area. Samples includes PC1 to PC5 in May
PR: the planting period in the restoration area. Samples includes PR1 to PR4 in May
LPC: the later period of in the control area. Samples includes LPC1 to LPC5 in August
LPR: the later period of in the restoration area. Samples includes LPR1 to LPR5 in August.
