# Supplementary material for: Variation in Bacterial Community Structures and Functions as Indicators of Response to the Restoration of Suaeda salsa: A Case Study of the Restoration in the Beidaihe Coastal Wetland
Source: Front Microbiol. 2022 Apr 25;13:783155. doi: 10.3389/fmicb.2022.783155 (PMC9082946; doi:10.3389/fmicb.2022.783155)
Supplement: Supplementary file 1 [file Data_Sheet_1.docx]

**Part 1**

**Identifying of key OTUs**

The random forest algorithm, proposed by L. Breiman in 2001, has been extremely successful as a general-purpose classification and regression method. The approach, which combines several randomized decision trees and aggregates their predictions by averaging, has shown excellent performance in settings where the number of variables is much larger than the number of observations (Biau et al., 2016). Moreover, it is versatile enough to be applied to many problems, such as ecological (Cutler et al., 2017; Xu et al., 2014), bioinformatics (Díaz-Uriarte et al., 2006) and so on.

In our study, a classification Random Forest analysis (Breiman 2001) was performed to assess the health of coastal wetland by randomForest package (Liaw et al., 2013). RandomForest package implements Breiman’s random forest algorithm (based on Breiman and Cutler’s original Fortran code) for classification and regression (Breiman et al., 2001). In the training data, the importance of each predictor variable (OTUs) is determined by evaluating the decrease in prediction accuracy (i.e., increase in the mean square error between observations and OOB predictions) when the data for that predictor are randomly permuted. This decrease is averaged over all trees to produce the final measure of importance (Wei et al. 2010). For example. The top 30 OTUs importance were showed in Fig 1.


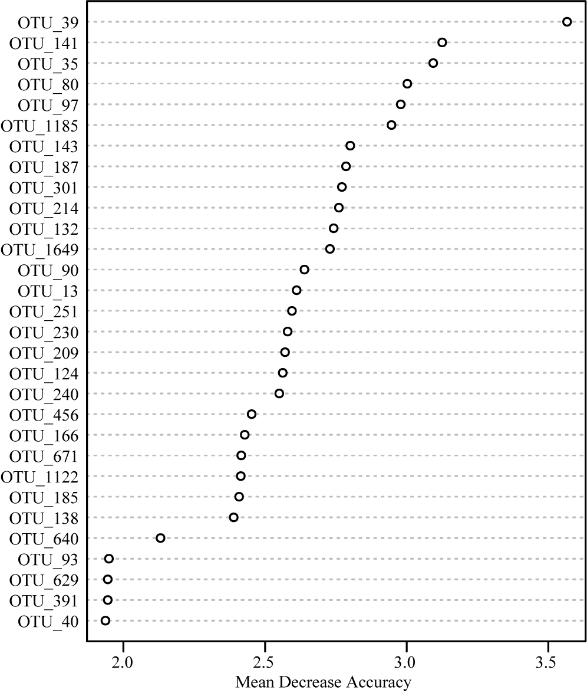


Fig. 1 Top 30 – variable (OTUs) importance in the training data

Establishing the final key OTUs

Among all variable (OTUs), not all OTUs in the RF model contributed to the classification of the model. Even, some not important OTUs can affect the classification results. To remove the non key OTUs, all OTUs ranked according to the importance (Mean Decrease Accuracy) from high to low and performed the 10-fold cross validation (repeat 5) (Fig. S2). We found that the top 30 important OTUs most contributed to classification of wetland health, which was chosen to the final key OTUs in our study.

**Reference:**

Biau, G., & Scornet, E. (2016). A random forest guided tour. Test, 25(2), 197-227.

Breiman, L. (2001). Random forests. Machine learning, 45(1), 5-32.

Cutler, D. R., Edwards Jr, T. C., Beard, K. H., Cutler, A., Hess, K. T., Gibson, J., & Lawler, J. J. (2007). Random forests for classification in ecology. Ecology, 88(11), 2783-2792.

Díaz-Uriarte, R., & Alvarez de Andrés, S. (2006). Gene selection and classification of microarray data using random forest. BMC bioinformatics, 7(1), 1-13.

Liaw, A., & Wiener, M. (2013). Documentation for R package randomForest. PDF). Retrieved, 15, 191.

Xu, Z., Malmer, D., Langille, M. G., Way, S. F., & Knight, R. (2014). Which is more important for classifying microbial communities: who’s there or what they can do?. The ISME journal, 8(12), 2357-2359.

Wei, C. L., Rowe, G. T., Escobar-Briones, E., Boetius, A., Soltwedel, T., Caley, M. J., et al. (2010). Global patterns and predictions of seafloor biomass using random forests. PloS one, 5(12), e15323.

**Part 2**

**Supplementary Figures**


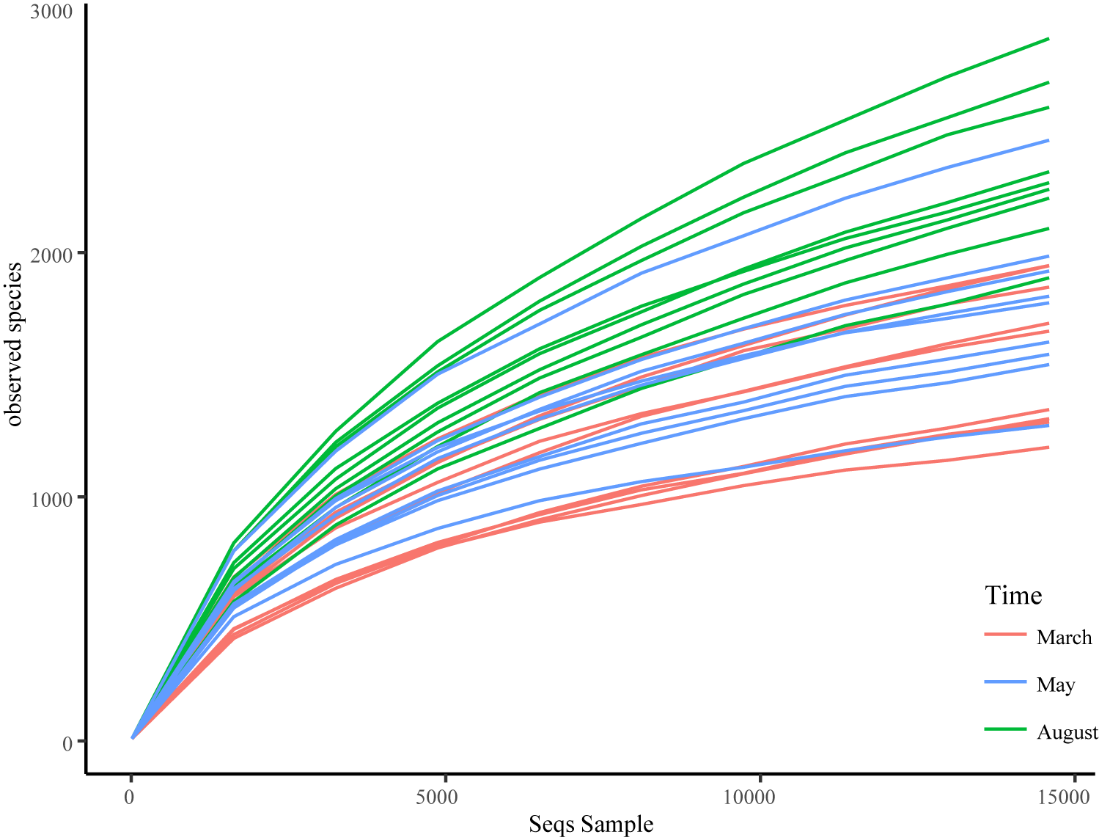


**Figure S1.** Rarefaction plots of 27 samples


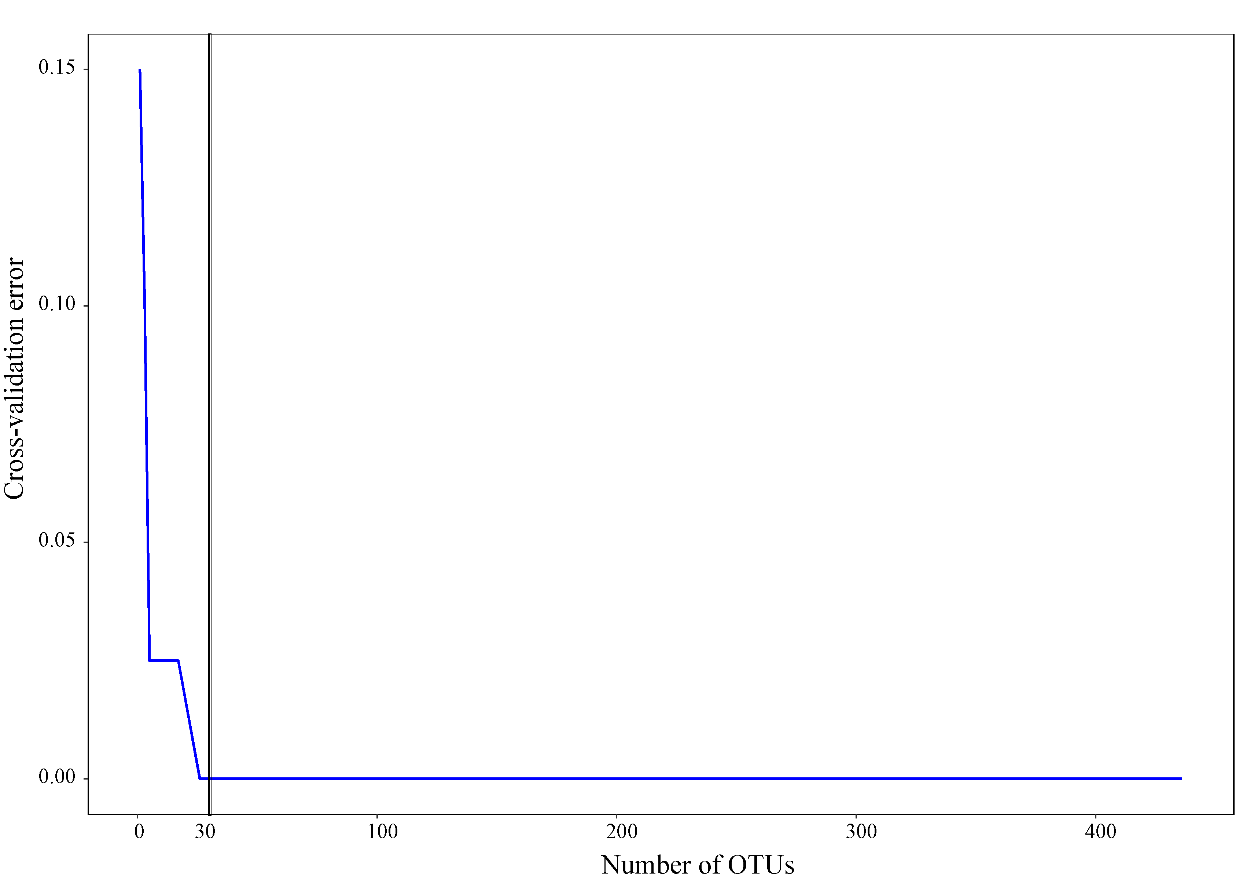
**Figure S2.** Ten-fold cross validation errors for random forest models based on their importance for model accuracy. A vertical line has been drawn on the x-axis at the point of including 30 of the most important OTUs discriminated wetland health.


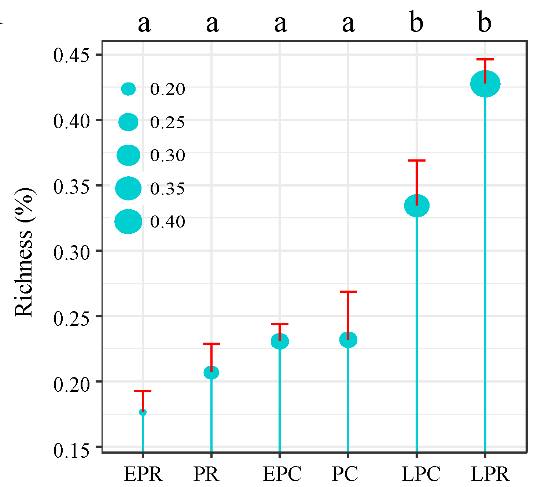
 **Figure S3.** The richness of bacterial community during the restoration.


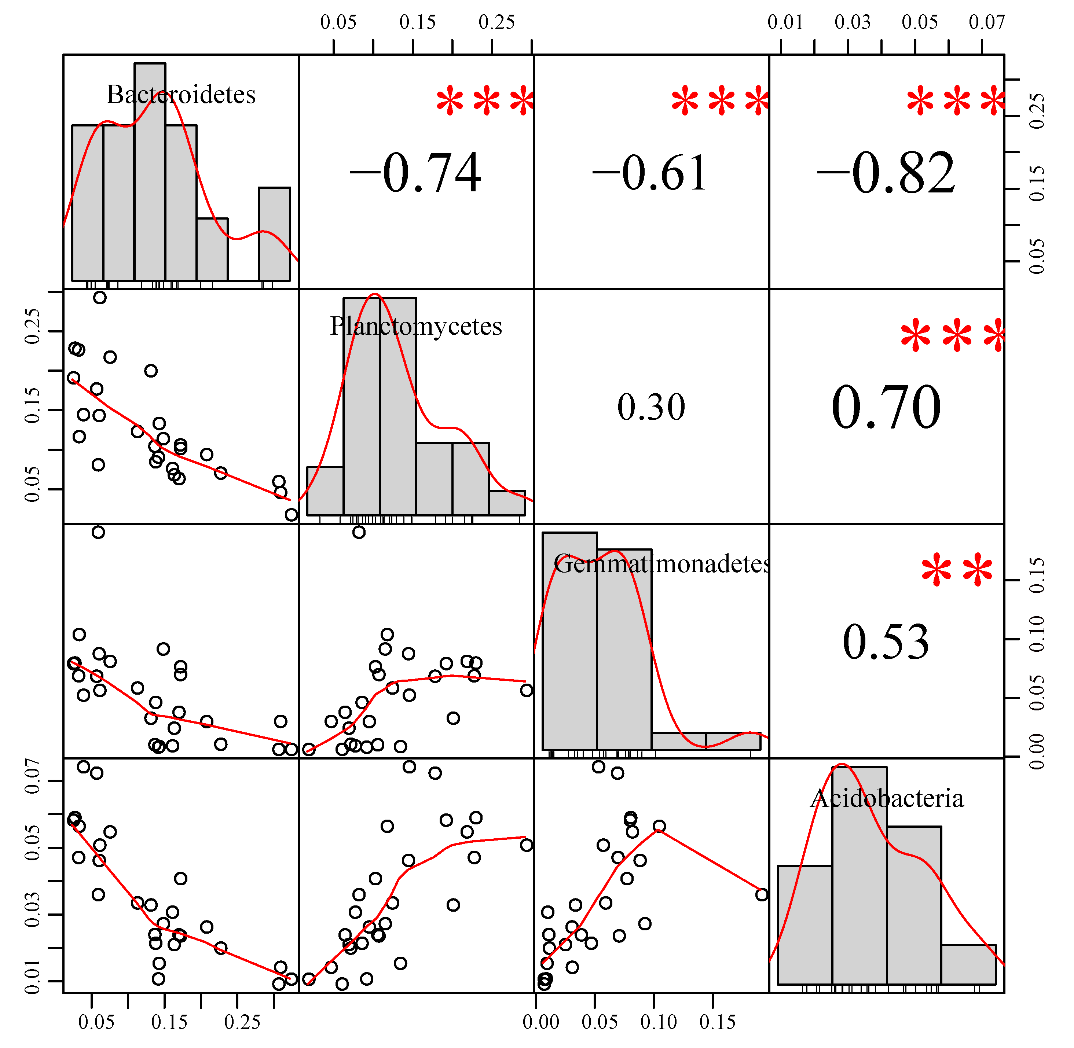


**Figure S4.** Correction analysis about the abundance of phyla during the restoration process. The number stands for Pearson correlation value. ⁎ , p < 0.05; ⁎ ⁎ , p < 0.01; ⁎ ⁎ ⁎ , p < 0.001.


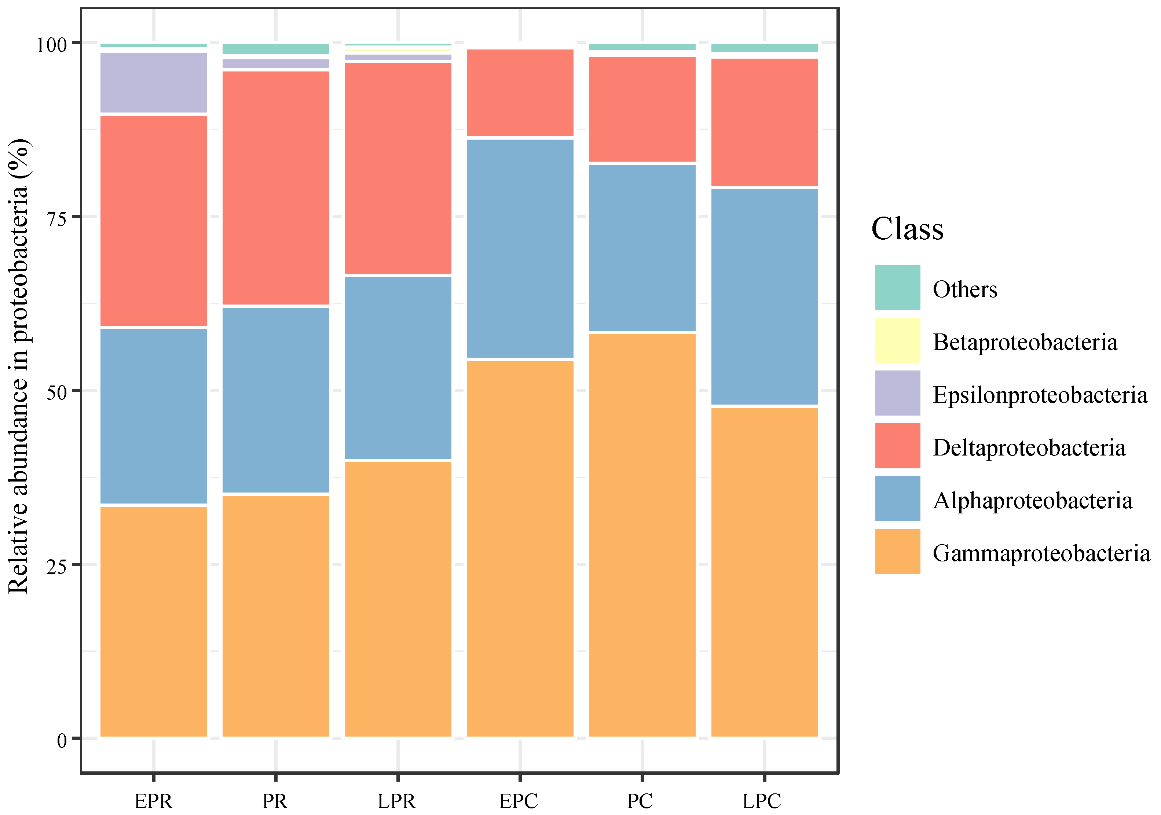
**Figure S5.** Bar plots of the dominant Class in Proteobacteria during different restoration periods in the degraded and non-degraded wetland.


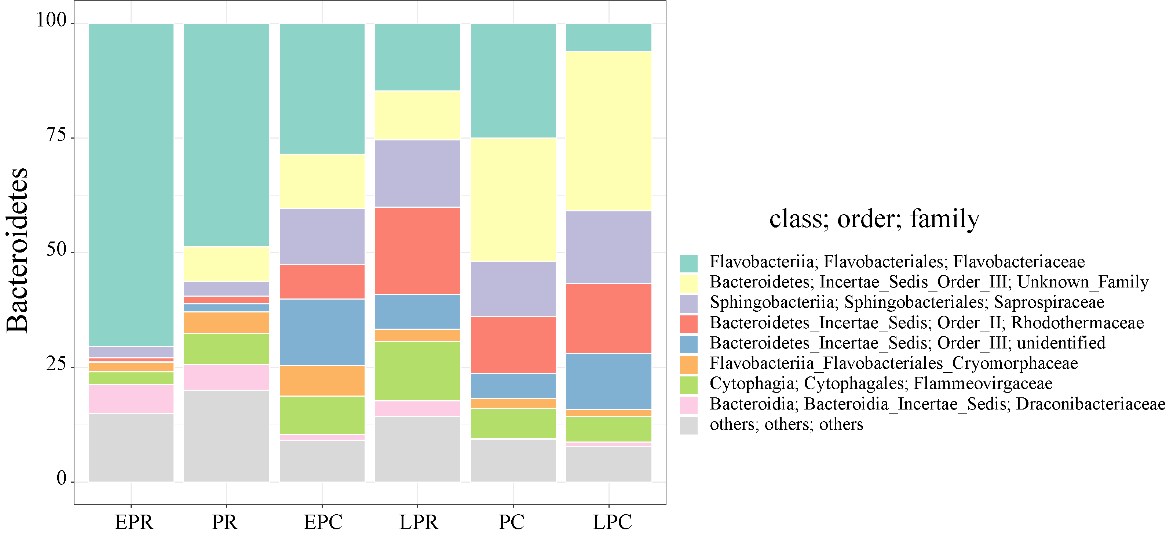


**Figure S6.** Bar plots of the dominant Class, Order, and Family in Bacteroidetes during different restoration periods in the degraded and non-degraded wetland.


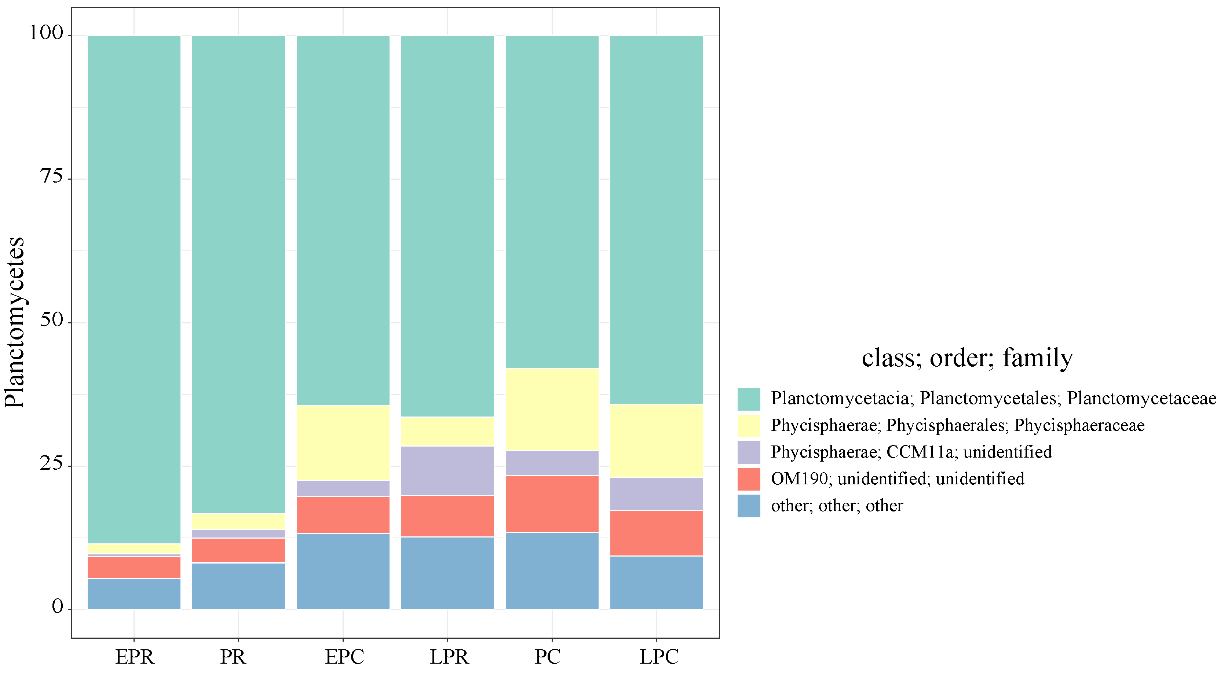


**Figure S7.** Bar plots of the dominant Class, Order, and Family in Planctomycetes during different restoration periods in the degraded and non-degraded wetland.


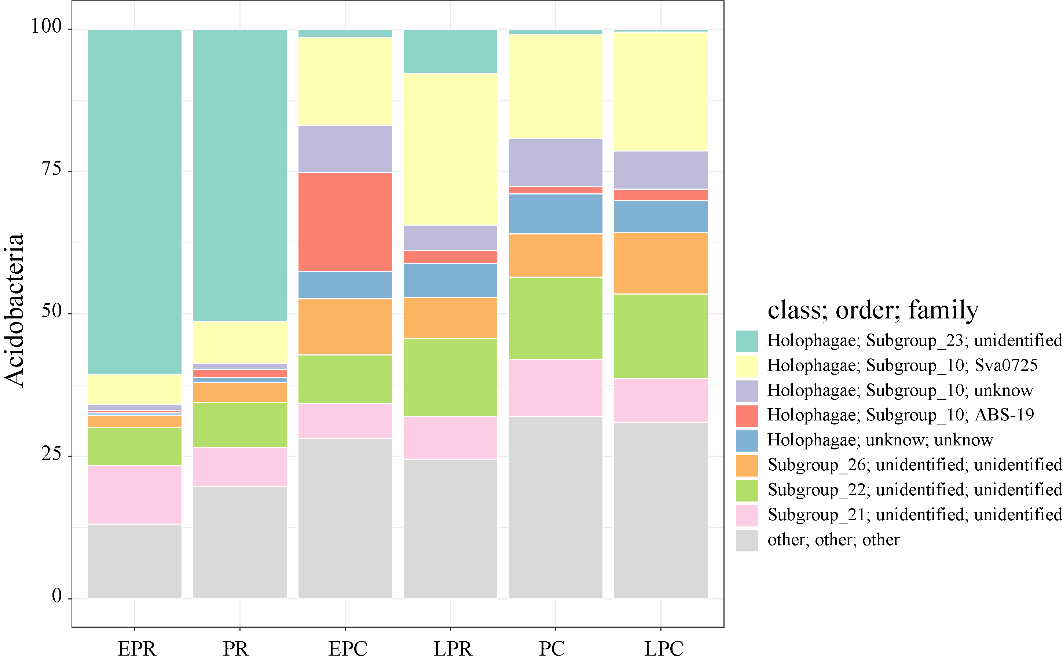


**Figure S8.** Bar plots of the dominant Class, Order, and Family in Acidobacteria during different restoration periods in the degraded and non-degraded wetland.


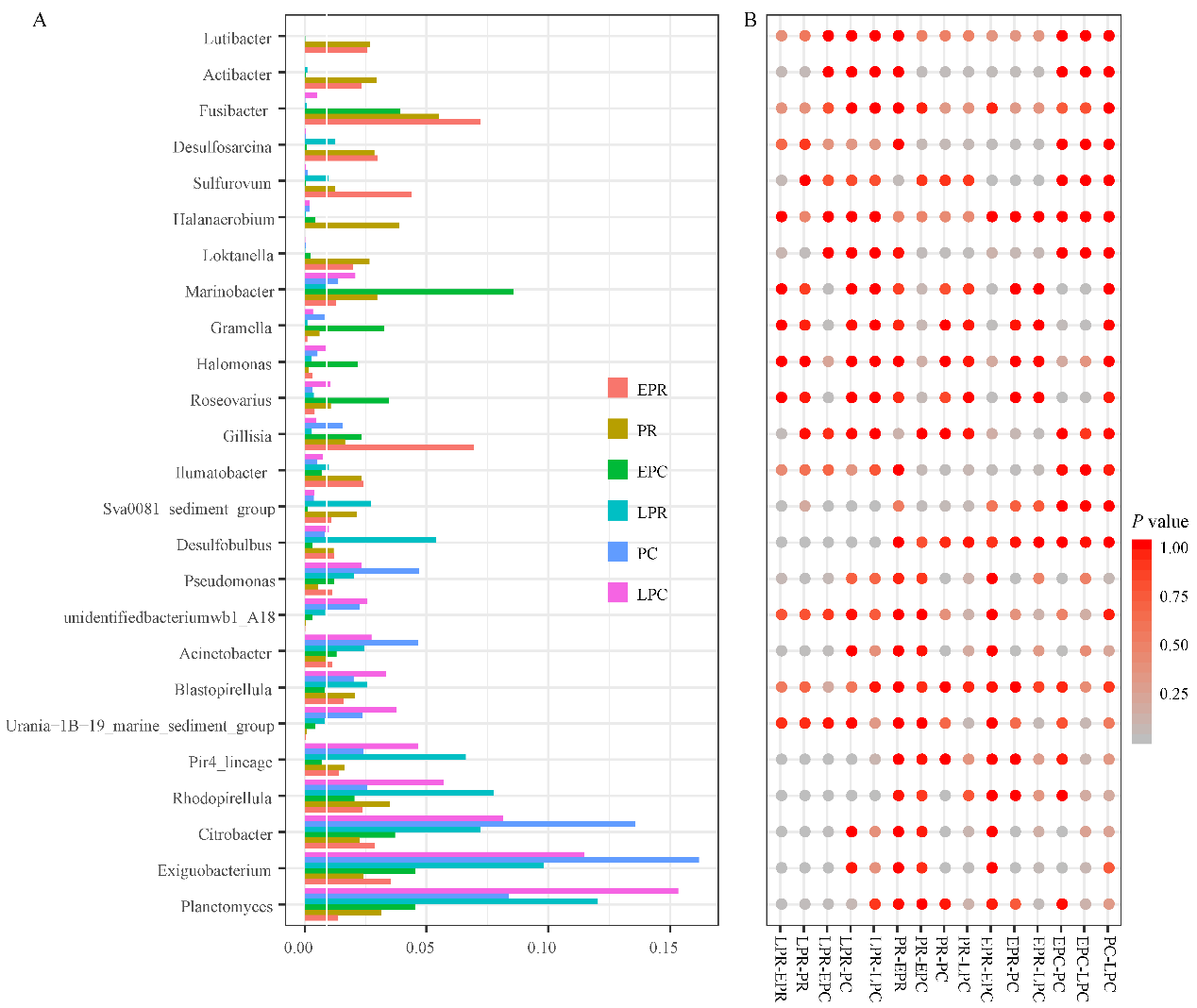


**Figure S9.** The dominant genus (A) and significantly different (B) between different periods.

**Supplementary Tables**

**Table S1.** The detailed information of samples in the Beidaihe coastal wetland.

| Groups | Month | region | Site | Sample ID |
| --- | --- | --- | --- | --- |
| EPR |  | Restoration area | R1 | EPR1 |
|  | March | Restoration area | R2 | EPR2 |
|  |  | Restoration area | R3 | EPR3 |
|  |  | Restoration area | R4 | EPR4 |
| PR |  | Restoration area | R1 | PR1 |
|  | May | Restoration area | R2 | PR2 |
|  |  | Restoration area | R3 | PR3 |
|  |  | Restoration area | R4 | PR4 |
| LPR |  | Restoration area | R1 | LPR1 |
|  |  | Restoration area | R2 | LPR2 |
|  | August | Restoration area | R3 | LPR3 |
|  |  | Restoration area | R4 | LPR4 |
| EPC |  | Control area | C1 | EPC1 |
|  |  | Control area | C2 | EPC2 |
|  | March | Control area | C3 | EPC3 |
|  |  | Control area | C4 | EPC4 |
|  |  | Control area | C5 | EPC5 |
| PC |  | Control area | C1 | PC1 |
|  |  | Control area | C2 | PC2 |
|  | May | Control area | C3 | PC3 |
|  |  | Control area | C4 | PC4 |
|  |  | Control area | C5 | PC5 |
| LPC |  | Control area | C1 | LPC1 |
|  |  | Control area | C2 | LPC2 |
|  | August | Control area | C3 | LPC3 |
|  |  | Control area | C4 | LPC4 |
|  |  | Control area | C5 | LPC5 |

**Table S2.** Valid data statistics of 16 S rRNA sequencing.

| Samples ID | Raw Tags | Clean Tags | The percentage of clean tags |
| --- | --- | --- | --- |
| EPC1 | 52154 | 50277 | 0.9640 |
| EPC2 | 49711 | 49014 | 0.9860 |
| EPC3 | 56519 | 54645 | 0.9668 |
| EPC4 | 53277 | 51718 | 0.9707 |
| EPC5 | 64448 | 62603 | 0.9714 |
| EPR1 | 52162 | 50575 | 0.9696 |
| EPR2 | 52889 | 49850 | 0.9425 |
| EPR3 | 54161 | 52873 | 0.9762 |
| EPR4 | 53587 | 52257 | 0.9752 |
| PC1 | 54117 | 53699 | 0.9923 |
| PC2 | 55182 | 54961 | 0.9960 |
| PC3 | 64844 | 63882 | 0.9852 |
| PC4 | 64816 | 64036 | 0.9880 |
| PC5 | 73055 | 71589 | 0.9799 |
| PR1 | 51735 | 50368 | 0.9736 |
| PR2 | 50571 | 49403 | 0.9769 |
| PR3 | 53333 | 52399 | 0.9825 |
| PR4 | 55234 | 53454 | 0.9678 |
| LPC1 | 106593 | 105215 | 0.9871 |
| LPC2 | 114700 | 113229 | 0.9872 |
| LPC3 | 103474 | 102768 | 0.9932 |
| LPC4 | 109550 | 108431 | 0.9898 |
| LPC5 | 110923 | 109793 | 0.9898 |
| LPR1 | 105704 | 103309 | 0.9773 |
| LPR2 | 117634 | 116119 | 0.9871 |
| LPR3 | 110335 | 108489 | 0.9833 |
| LPR4 | 104457 | 103381 | 0.9897 |

**Table S3**. The result of prediction of wetland health in the testing data.

| Classification | Predict classification | |  |
| --- | --- | --- | --- |
|  | Degeneration | No-degeneration | Total |
| Degeneration | 3 | 1 | 4 |
| No- degeneration | 4 | 11 | 15 |
| Total | 7 | 12 | 19 |

| **Table. S****4** The annotated message of 30 key OTUs for the assessment of wetland health. | | | | | |
| --- | --- | --- | --- | --- | --- |
| id | phylum | class | order | family | genus |
| OTU_391 | Bacteroidetes | Flavobacteriia | Flavobacteriales | Flavobacteriaceae | Winogradskyella |
| OTU_143 | Proteobacteria | Gammaproteobacteria | Alteromonadales | Alteromonadaceae | Marinobacter |
| OTU_132 | Firmicutes | Clostridia | Clostridiales | Family_XII | Fusibacter |
| OTU_39 | Bacteroidetes | Flavobacteriia | Flavobacteriales | Flavobacteriaceae | Sediminicola |
| OTU_35 | Proteobacteria | Gammaproteobacteria | Cellvibrionales | Halieaceae | Halioglobus |
| OTU_97 | Bacteroidetes | Flavobacteriia | Flavobacteriales | Flavobacteriaceae | Flavobacterium |
| OTU_456 | Planctomycetes | Phycisphaerae | Phycisphaerales | Phycisphaeraceae | marine_sediment_group |
| OTU_93 | Gemmatimonadetes | AKAU4049 | unidentified | unidentified | unidentified |
| OTU_90 | Proteobacteria | Alphaproteobacteria | Rhodospirillales | Rhodospirillaceae | unidentified |
| OTU_640 | Latescibacteria | unidentified | unidentified | unidentified | unidentified |
| OTU_301 | Proteobacteria | Gammaproteobacteria | Xanthomonadales | unidentified | unidentified |
| OTU_251 | Gemmatimonadetes | PAUC43f_marine_benthic_group | unidentified | unidentified | unidentified |
| OTU_230 | Proteobacteria | Gammaproteobacteria | Gammaproteobacteria_Incertae_Sedis | Unknown_Family | unidentified |
| OTU_214 | SBR1093 | unidentified | unidentified | unidentified | unidentified |
| OTU_209 | Proteobacteria | Alphaproteobacteria | Rhodospirillales | MSB-1E8 | unidentified |
| OTU_185 | Proteobacteria | Alphaproteobacteria | Rhodospirillales | Rhodospirillaceae | unidentified |
| OTU_166 | Proteobacteria | Gammaproteobacteria | unidentified | unidentified | unidentified |
| OTU_1649 | Gemmatimonadetes | BD2-11_terrestrial_group | unidentified | unidentified | unidentified |
| OTU_138 | Bacteroidetes | Bacteroidetes_Incertae_Sedis | Order_II | Rhodothermaceae | unidentified |
| OTU_124 | Chloroflexi | Anaerolineae | Anaerolineales | Anaerolineaceae | unidentified |
| OTU_1185 | Acidobacteria | Subgroup_26 | unidentified | unidentified | unidentified |
| OTU_1122 | Gemmatimonadetes | BD2-11_terrestrial_group | unidentified | unidentified | unidentified |
| OTU_80 | Proteobacteria | Deltaproteobacteria | Desulfobacterales | Desulfobacteraceae | Sva0081_sediment_group |
| OTU_671 | Proteobacteria | Deltaproteobacteria | Desulfobacterales | Desulfobacteraceae | Sva0081_sediment_group |
| id | phylum | class | order | family | genus |
| OTU_629 | Planctomycetes | Planctomycetacia | Planctomycetales | Planctomycetaceae | Rhodopirellula |
| OTU_13 | Planctomycetes | Planctomycetacia | Planctomycetales | Planctomycetaceae | Planctomyces |
| OTU_40 | Proteobacteria | Alphaproteobacteria | Rhodospirillales | Rhodospirillaceae | unidentified |
| OTU_240 | Proteobacteria | Alphaproteobacteria | Rhodobacterales | Rhodobacteraceae | unidentified |
| OTU_187 | Acidobacteria | Holophagae | unidentified | unidentified | unidentified |
